# Biological and psychosocial risk factors for psychotic major depression

**DOI:** 10.1007/s00127-015-1131-1

**Published:** 2015-10-31

**Authors:** M. Heslin, R. Desai, J. M. Lappin, K. Donoghue, B. Lomas, U. Reininghaus, A. Onyejiaka, T. Croudace, P. B. Jones, R. M. Murray, P. Fearon, G. A. Doody, P. Dazzan, H. L. Fisher, A. Demjaha, T. Craig, C. Morgan

**Affiliations:** Institute of Psychiatry, Psychology and Neuroscience, King’s College London, De Crespigny Park, Denmark Hill, London, SE5 8AF UK; University of New South Wales, Sydney, Australia; University of Nottingham, Nottingham, UK; Maastricht University, Maastrict, The Netherlands; University of Dundee, Dundee, UK; University of Cambridge, Cambridge, UK; National Institute for Health Research (NIHR), Mental Health Biomedical Research Centre at South London and Maudsley NHS Foundation Trust, London, UK; Trinity College, Dublin, Ireland

**Keywords:** Depression, Epidemiology, Psychosis, Risk factors

## Abstract

**Aims:**

Few studies have investigated risk factors for psychotic major depression (PMD). We aimed to investigate the biological and psychosocial risk factors associated with PMD compared with other psychotic disorders.

**Methods:**

Based on the aetiology and ethnicity in schizophrenia and other psychoses (ÆSOP) study, we used a case–control study to identify and recruit, at baseline and 10-year follow-up, all first episode cases of psychosis, presenting for the first time to specialist mental health services in defined catchment areas in the UK. Population-based controls were recruited from the same areas. Data were collected on: sociodemographics; social isolation; childhood adversity; life events; minor physical anomalies; and neurological soft signs.

**Results:**

Living alone (aOR = 2.26, CI = 1.21–4.23), basic level qualification (aOR = 2.89, CI = 1.08–7.74), being unemployed (aOR = 2.12, CI = 1.13–3.96), having contact with friends less than monthly (aOR = 4.24, CI = 1.62–11.14), having no close confidants (aOR = 4.71, CI = 2.08–10.68), having experienced childhood adversity (aOR = 2.57, CI = 1.02–6.44), family history of mental illness (aOR = 10.68, CI = 5.06–22.52), family history of psychosis (aOR = 12.85, CI = 5.24–31.51), and having more neurological soft signs (aOR = 1.15, CI = 1.07–1.24) were all associated with a follow-up diagnosis of PMD and schizophrenia. Few variables associated with PMD were also associated with a diagnosis of bipolar disorder. Minor physical anomalies were associated with a follow-up diagnosis of schizophrenia and bipolar disorder, but not PMD.

**Conclusions:**

Risk factors associated with PMD appear to overlap with those for schizophrenia, but less so for bipolar disorder. Future work on the differential aetiology of PMD, from other psychoses is needed to find the ‘specifier’ between PMD and other psychoses. Future research on aetiology in PMD, and perhaps other psychoses, should account for diagnostic change.

**Electronic supplementary material:**

The online version of this article (doi:10.1007/s00127-015-1131-1) contains supplementary material, which is available to authorized users.

## Introduction

An understanding of the risk factors involved in mental disorders may inform the development of more effective interventions [12] or even prevention [20]. Risk factors in psychosis are commonly broken down into biological and psychosocial, with psychosocial risk factors often being underestimated [40]. However, many researchers have highlighted the importance of the social environment in the aetiology of psychosis [2] and the potential for psychosocial factors to be used in designing interventions to treat and prevent disorder [14, 29, 39].

ICD–10 [53] classifies a depressive disorder with the addition of delusions, hallucinations or depressive stupor as a severe depressive episode with psychotic symptoms; this is also known as psychotic major depression (PMD). PMD is a largely under-researched disorder [10] and is commonly excluded from risk studies [48]; the majority of studies of risk factors in psychosis focus on schizophrenia and bipolar disorder [34]. Perhaps PMD is understudied as it is viewed as a relatively rare disorder, or perhaps as it is viewed as an unstable diagnosis which is likely to change.

Indeed, only two studies have investigated psychosocial risk factors in PMD cases. Fisher [15] used data from the ÆSOP (aetiology and ethnicity in schizophrenia and other psychoses) first episode psychosis study and reported increased prevalence of severe childhood maternal physical abuse (OR 3.81, CI 1.07–13.60), childhood maternal separation (OR 1.97, CI 0.78–4.97) and childhood sexual abuse (OR 1.82, CI 0.56–5.91) in those with PMD, compared with controls. However, these increases were not statistically significant once potential confounding was accounted for, with the odds ratio for maternal physical abuse and maternal separation decreasing to OR 1.94 (CI 0.30–12.67) and OR 1.08 (CI 0.31–3.70), respectively. Fisher did not examine other forms of adversity (e.g. neglect) nor whether exposure to ‘any’ form of childhood adversity might be associated with PMD. Samuel and Varghese investigated life events in a randomly selected sample of patients with PMD from an outpatient clinic in India [47]. They found that life events prior to onset were reported in 53 % of patients. Unfortunately, the authors did not include a control group and 60 % of the PMD cases had a history of bipolar disorder, bringing into question the validity of their diagnosis as PMD cases.

The above studies used direct measures of social experience; others have examined markers of social position and context. These include sociodemographics (e.g. age, gender, ethnicity) and educational attainment and have found that PMD is more common in women [1, 17, 18, 45], increases with age [10, 46, 48] and is elevated in Black African and Black Caribbean migrants and their descendants [30]. Jeste et al. [28] reported no differences in educational attainment between PMD and schizophrenia and non-psychotic major depression. No studies have examined social isolation or unemployment as risk factors for PMD specifically, but social isolation has been associated with psychosis in general [13, 41]—ÆSOP study; [50] and with depression [50], and unemployment has been associated with psychosis in general [13, 43]—ÆSOP study).

With the dominance of the biopsychosocial model of aetiology of psychosis, any investigation of risk factors involved in psychotic disorder would be unwise to ignore the role of biological factors. Minor physical anomalies (MPAs) are thought to be indicators of altered morphogenesis during the first or early second trimester in utero and act as a biological marker of developmental disturbance [52]. Increased prevalence of MPAs has been documented in schizophrenia [22, 23, 35] but not investigated in PMD. Similarly, neurological soft signs (NSS) are thought to indicate developmental disturbances, and have been associated with schizophrenia but not investigated in PMD [11], ÆSOP study). Finally, family history is an indicator of genetic risk that has not until now been investigated in PMD.

As well as there being a limited amount of information on the aetiology of PMD, there are also major methodological limitations with the studies to date. While first episode studies are, in many ways, optimal for studying aetiology, this can be problematic when investigating specificity for disorder, as diagnoses are often inaccurate at this point [6]. Heslin et al. (2015; ÆSOP study) compared baseline and lifetime (determined at 10-year follow-up) diagnoses in the ÆSOP study and reported that only 47 % (*n* = 26) of those diagnosed with PMD at baseline had the same diagnosis at follow-up. Therefore, studies which only investigate risk factors in relation to baseline diagnoses may be inaccurate. Furthermore, there is the issue of sampling. The majority of studies on risk factors (and some of those mentioned above) are based on non-first episode samples. Cases recruited from non-first episode samples are effectively sampling prevalence cases in treatment, biasing the investigation towards those who are more unwell and excluding others who have recovered.

This study used baseline and follow-up data from the ÆSOP study to investigate the psychosocial and biological risk factors associated with PMD compared with other psychotic diagnostic groups (i.e. non-affective psychoses, bipolar disorder with psychotic symptoms) using diagnoses established at first contact with services and at 10-year follow-up.

## Methods

### Baseline

#### Setting

This paper is based on data from the ÆSOP programme [31, 42], which comprises an incidence, case control, and 10-year follow-up study of all individuals with a first episode of psychosis presenting for the first time to specialist mental health services (adult community mental health teams, inpatient units, forensic services, learning disability services, adolescent mental health services, and drug and alcohol units) between September 1997 and August 1999 in defined catchment areas of Nottingham and London (baseline). First episode was defined as the first contact with specialist services for psychosis.

#### Cases

Within tightly defined geographical areas, all cases who presented to specialist mental health services were screened for psychosis using the Screening Schedule for Psychosis [26] completed using information from clinical notes and corroboration from mental health staff and, where possible, by interview with the participant. All cases who were experiencing a first episode of psychosis [codes F20–29 and F30–33 in ICD–10 [53]] were included in the incidence study, but only cases with F20 or F30-33 are included in this paper. Each person identified for the incidence study was invited to participate in the case–control section of the study.

Inclusion criteria for cases were: aged between 16 and 64 years inclusive with a first episode of psychosis. Exclusion criteria for cases were: evidence of psychotic symptoms precipitated by an organic cause; transient psychotic symptoms resulting from acute intoxication as defined by ICD–10 [53]; previous contacts with mental health services for psychosis; and moderate or severe learning difficulties, or an IQ of less than 50 [53].

#### Controls

A population-based sample of controls without a history of psychosis was recruited using the UK postal address file (PAF) [27]. The PAF was used to generate a random sample of 10 target addresses that were within the same postcode area as each of the cases. This ensured controls were selected from the same geographic location as cases. Target addresses were visited by researchers on three separate occasions, at different times of day and on different days to ensure maximum likelihood of contact being made and to minimise sampling bias. If there were no responses from the address or if there was a refusal from occupants, then the next target address was approached. A Kish [32] grid was used to randomly select one control if more than one eligible control was available from each household.

Exclusion criteria for controls were: current or past psychotic disorder; previous contacts with mental health services for psychosis; aged less than 16 years or over 65 years; insufficient level of English to complete the interviews; and moderate or severe learning difficulties, or an IQ of less than 50 [53].

#### Measures

Using all possible sources of information (interview, case records, informants), data were collected at baseline on: age at interview, gender, ethnicity (grouped into White British, African-Caribbean, Black African, White Other, Asian and ‘Other’), place of birth (UK born versus born outside of the UK), employment [unemployed versus employed and other (student, full time parent, housewife/house husband)], education (basic versus further versus higher), and social isolation variables [relationship status (stable relationship versus single), living circumstances (with people versus alone), contact with friends (daily to monthly versus less than monthly), contact with family (daily to monthly versus less than monthly) and close confidants (yes versus no; defined by asking ‘do you have someone you can confide in?’)] using the Medical Research Council Socio-demographic Schedule (MRC-SDS) [36]. Childhood adversity was assessed using the Childhood Experience of Care and Abuse Questionnaire (CECA-Q) [3]. The CECA-Q is a questionnaire based on a semi-structured interview and is designed to measure childhood experiences of neglect, antipathy, physical abuse and sexual abuse reported retrospectively. Data from the CECA-Q were used to create an ‘any adversity’ variable (see [16] for details). Exposure to severe life events in the year prior to onset of psychosis was assessed and defined using the Life Events and Difficulties Schedule (LEDS) [7, 8]. The LEDS (LEDS) is a semi-structured interview which is used to gather information on the presence or absence of a range of stressful life events and on-going difficulties. The LEDS is based on detailed definitions of what should and should not be included as an event or difficulty. This helps to ensure consistency between different studies but also guards against investigator bias that might arise were the inclusion of incidents determined retrospectively by the investigator.

Family history of psychosis, and family history of any mental illness was ascertained using the Family Interview for Genetics (FIGS; [37]). The FIGS is a schedule for gathering diagnostic information about relatives of a proband within a study and is made up of three parts: the family tree; general screening questions, and the symptom checklists. The family tree is used to orientate the interviewee to who is included. The screening questions are then used to gather information on possible mental illness in first-degree relatives. The symptom checklists are then used to elicit specific information about relatives who have an indication of mental illness from the screening questions. Within this study, a shortened version of the FIGS was used including the family tree, the general screening questions, and only the depression, mania and psychosis symptom checklists. Minor physical anomalies were assessed using the Lane scale [33]. The Lane scale contains qualitative measurements of the head and face designed to identify anomalies of facial symmetry and details of eye, ear, nose, mouth, palate and hairline morphology.

Minor physical anomalies (MPAs) are present in developmental disorders and may be indicators of

ectodermal abnormalities that occur in the developing foetus. Neurological soft signs were assessed using an expanded version of the Neurological Evaluation Scale [9, 21]. The Neurological Evaluation Scale is a structured clinical evaluation intended to assess neurological impairments that have been reported to have increased prevalence in schizophrenia.

The Schedules for Clinical Assessment in Neuropsychiatry [SCAN version 2; [54]] was used to elicit symptom-related data at the time of presentation. The SCAN incorporates the Present State Examination Version 10, to determine whether a range of symptoms are present, and how severe they are. These symptoms are part of a comprehensive and defined list. A shortened version of the SCAN was used to focus solely on symptoms of depression, mania and psychosis. ICD-10 [53] psychosis diagnoses were determined using all available clinical information (excluding clinical diagnosis) on the basis of consensus meetings involving at least one of the principal investigators with other members of the research team. This was made as soon as possible after first contact (generally within a few weeks). Diagnoses were made blind to ethnicity.

All measures have been previously validated and were collected by trained, experienced mental health research workers. The SCAN, Lane Scale and Neurological Evaluation Scale were collected by a qualified psychiatrist, specifically trained in these measures.

### Follow-up

Cases were followed up 10 years after first contact with services. In brief, we made contact with cases who were still in contact with mental health services, through those services. For those who were not, we sent letters to their last known address and, if necessary, we made a maximum of three visits to their address (morning, afternoon and evening). For those who had moved address, and for whom we had general practitioner (GP) contact details, we sought to make contact and invite them to participate through their GP (See [42] for full details). The WHO Life Chart [24, 51] was completed for each case using clinical interview and case notes, to map course of illness and symptom history. The SCAN was also completed in relation to the preceding month where possible. Follow-up diagnosis using a consensus approach was based on all this clinical information, and blind to ethnicity and baseline diagnosis.

### Ethics

Ethical approval was granted by the Institute of Psychiatry and South London and Maudsley (SLaM) Research Ethics Committee and by the North Nottingham Healthcare NHS Trust Ethics Committee.

### Analyses

All data were analysed using STATA 10 [49]. Differences in missing data between cases and controls, and between baseline diagnostic groups were compared using Chi-square tests. Multinomial logistic regression was used to estimate odds ratios as this allows for the comparison of each diagnostic group to the control group within a single regression model. Cases with missing data were automatically dropped from each analysis by STATA. All regression models were adjusted for age, gender, study centre and ethnicity.

## Results

At baseline, a total of 557 first episode cases were identified. Data presented here are based on the incidence sample collected over the first 2 years (excluding: non-incidence cases collected for the brain imaging component of the study; cases oversampled in the third year to increase the numbers for the ethnicity component of the study; and cases excluded post baseline). This led to a total number of 505 cases: 304 from London and 201 from Nottingham. Data presented here are for the PMD, schizophrenia and bipolar disorder cases only; therefore, the total cases included in this paper is 360. A total of 391 controls were recruited: 183 from London and 208 from Nottingham.

### Sample characteristics

Of the analytic sample of 360 cases (Table 1), 72 (14.3 %) cases had a diagnosis of PMD, 218 (43.2 %) had a diagnosis of schizophrenia and 70 (13.9 %) had a diagnosis of bipolar disorder. PMD cases had a median age of 32.5 years (range 16–61 years), 50.0 % were women, 48.6 % were from London, and 51.4 % were white British.

**Table 1:** Sample characteristics at baseline for cases and controls based on baseline diagnosis and follow-up diagnosis

Baseline diagnosis	Controls (*n* = 391)	PMD (*n* = 72)	Schizophrenia (*n* = 218)	Bipolar (*n* = 70)
	*Median (IQR)*	*Median (IQR)*	*Median (IQR)*	*Median (IQR)*
Age	35.00 (28–47)	32.50 (25–41)	29.00 (22–35)	27.00 (23–33)

	*N* (%)	*N* (%)	*N* (%)	*N* (%)
Study centre				
London	183 (46.8)	35 (48.6)	151 (69.3)	44 (62.9)
Nottingham	208 (53.2)	37 (51.4)	67 (30.7)	26 (37.1)
Gender				
Male	161 (41.2)	36 (50.0)	140 (64.2)	33 (47.1)
Female	230 (58.8)	36 (50.0)	78 (35.8)	37 (52.9)
Ethnicity				
White British	241 (61.6)	37 (51.4)	81 (37.2)	27 (38.6)
African-Caribbean	74 (18.9)	8 (11.1)	61 (28.0)	14 (20.0)
Black African	21 (5.4)	7 (9.7)	33 (15.1)	11 (15.7)
White Other	42 (10.7)	4 (5.6)	22 (10.9)	4 (5.7)
Asian	8 (2.0)	7 (9.7)	10 (4.6)	6 (8.6)
Other (all)	5 (1.3)	9 (12.5)	11 (5.1)	8 (11.4)

Follow-up diagnosis	Controls (*n* = 391)	PMD (*n* = 51)	Schizophrenia (*n* = 225)	Bipolar (*n* = 73)
	*Median (IQR)*	*Median (IQR)*	*Median (IQR)*	*Median (IQR)*
Age	35.00 (28–47)	36.00 (30–46)	28.00 (22–35)	27.00 (23–33)

	*N* (%)	*N* (%)	*N* (%)	*N* (%)
Study centre				
London	183 (46.8)	22 (43.1)	155 (68.9)	43 (58.9)
Nottingham	208 (53.2)	29 (56.9)	70 (31.1)	30 (41.1)
Gender				
Male	161 (41.2)	25 (49.0)	144 (64.0)	27 (37.0)
Female	230 (58.8)	26 (51.0)	81 (36.0)	46 (63.0)
Ethnicity				
White British	241 (61.6)	31 (60.8)	87 (38.7)	33 (45.2)
African-Caribbean	74 (18.9)	6 (11.8)	66 (29.3)	17 (23.3)
Black African	21 (5.4)	3 (5.9)	34 (15.1)	9 (12.3)
White Other	42 (10.7)	3 (5.9)	18 (8.0)	4 (5.5)
Asian	8 (2.0)	4 (7.8)	8 (3.6)	6 (8.2)
Other (all)	5 (1.3)	4 (7.8)	12 (5.3)	4 (5.5)

*IQR* interquartile range, *PMD* psychotic major depression

Of the 505 patients included at baseline in this study, 79.8 % (403) had sufficient information to make a follow-up diagnosis based on at least 8 years of information, therefore 20.2 % were lost to follow-up, and were excluded from the lifetime diagnosis analyses. At follow-up, 51 (12.7 %) cases had a diagnosis of PMD, 225 (55.8 %) had a diagnosis of schizophrenia, and 73 (18.1 %) had a diagnosis of bipolar disorder (see Heslin et al. [25] for further details).

### Missing data

As shown in Table 2, there was a significant difference (in terms of size and statistical significance) between cases and controls in terms of missing data in every risk factor variable (controls 0–68.3 % missing; cases 0–80.6 % missing).

**Table 2:** Missing data by case–control status (baseline diagnosis)

	Controls (*n* = 391)	Cases (*n* = 360)	*χ* ^2^, *df*	*P* value
	*N* (%)	*N* (%)		
Age				
Present	391 (100)	360 (100)	–	–
Missing	0 (0)	0 (0)		
Study centre				
Present	391 (100)	360 (100)	–	–
Missing	0 (0)	0 (0)		
Gender				
Present	391 (100)	360 (100)	–	–
Missing	0 (0)	0 (0)		
Ethnicity				
Present	391 (100)	360 (100)	–	–
Missing	0 (0)	0 (0)		
Place of birth				
Present	391 (100)	353 (98.1)	7.7, 1	0.006
Missing	0 (0)	7 (1.9)		
Living circumstances				
Present	391 (100)	356 (98.9)	4.4, 1	0.037
Missing	0 (0)	4 (1.1)		
Relationship status				
Present	391 (100)	345 (95.8)	16.6, 1	<0.001
Missing	0 (0)	15 (4.2)		
Ever relationship				
Present	386 (98.7)	267 (74.2)	99.6, 1	<0.001
Missing	5 (1.3)	93 (25.8)		
Level of education				
Present	388 (99.2)	350 (97.2)	4.5, 1	0.035
Missing	3 (0.8)	10 (2.8)		
Employment status				
Present	391 (100)	349 (96.9)	12.1, 1	<0.001
Missing	0 (0)	11 (3.1)		
Contact with friends				
Present	378 (96.7)	237 (65.8)	120.2, 1	<0.001
Missing	13 (3.3)	123 (34.2)		
Contact with family				
Present	368 (94.1)	241 (66.9)	90.3, 1	<0.001
Missing	23 (5.9)	119 (33.1)		
Close confidants				
Present	388 (99.2)	277 (76.9)	90.8, 1	<0.001
Missing	3 (0.8)	83 (23.1)		
Life events				
Present	147 (37.6)	70 (19.4)	30.1, 1	<0.001
Missing	244 (62.4)	290 (80.6)		
Childhood adversity				
Present	242 (61.9)	137 (38.1)	42.6, 1	<0.001
Missing	149 (38.1)	223 (61.9)		
Family history of any mental illness				
Present	276 (72.4)	391 (100)	124.719, 1	<0.001
Missing	105 (27.6)	0 (0)		
Family history of psychosis				
Present	276 (72.4)	391 (100)	124.719, 1	<0.001
Missing	105 (27.6)	0 (0)		
NSS				
Present	126 (32.2)	191 (53.1)	33.34, 1	<0.001
Missing	265 (67.8)	169 (46.9)		
MPAs				
Present	124 (31.7)	172 (47.8)	20.26, 1	<0.001
Missing	267 (68.3)	188 (52.2)		

*df* degrees of freedom, *MPA* minor physical abnormalities, *NSS* neurological soft signs, *PMD* psychotic major depression

Table 3 shows the comparison of missing data between the PMD, schizophrenia and bipolar disorder groups. There were significant differences (in terms of size and statistical significance) between the diagnostic groups in terms of missing data on the following variables, with schizophrenia cases having the most missing data on every variable: ever in a relationship (between 14.3 and 31.7 % missing); contact with family (between 21.4 and 39.5 % missing); contact with friends (between 24.3 and 39.5 % missing); life events (between 70.8 and 84.4 % missing); childhood adversity (between 41.4 and 67.9 % missing); neurological soft signs (between 27.1 and 56.9 % missing); and minor physical anomalies (between 30.0 and 63.3 % missing). This issue is explored in the discussion.

**Table 3:** Missing data by diagnostic (baseline diagnosis) group (*n* = 360)

	PMD (*n* = 72)	Schizophrenia (*n* = 218)	Bipolar (*n* = 70)	*χ* ^2^, *df*	*P* value
	*N* (%)	*N* (%)	*N* (%)		
Age					
Present	72 (100)	218 (100)	70 (100)	–	–
Missing	0 (0)	0 (0)	0 (0)		
Study centre					
Present	72 (100)	218 (100)	70 (100)	–	–
Missing	0 (0)	0 (0)	0 (0)		
Gender					
Present	72 (100)	218 (100)	70 (100)	–	–
Missing	0 (0)	0 (0)	0 (0)		
Ethnicity					
Present	72 (100)	218 (100)	70 (100)	–	–
Missing	0 (0)	0 (0)	0 (0)		
Place of birth					
Present	72 (100)	212 (97.3)	69 (98.6)	2.3, 2	0.321
Missing	0 (0)	6 (2.8)	1 (1.4)		
Living circumstances					
Present	70 (97.2)	216 (99.1)	70 (100)	2.7, 2	0.262
Missing	2 (2.8)	2 (0.9)	0 (0)		
Relationship status					
Present	69 (95.8)	206 (94.5)	70 (100)	4.0, 2	0.134
Missing	3 (4.2)	12 (5.5)	0 (0)		
Ever relationship					
Present	58 (80.6)	149 (68.3)	60 (85.7)	10.3, 2	0.006
Missing	14 (19.4)	69 (31.7)	10 (14.3)		
Level of education					
Present	69 (95.8)	212 (97.3)	69 (98.6)	1.0, 2	0.611
Missing	3 (4.2)	6 (2.8)	1 (1.4)		
Employment status					
Present	70 (97.2)	209 (95.9)	70 (100)	3.1, 2	0.215
Missing	2 (2.8)	9 (4.1)	0 (0)		
Contact with friends					
Present	52 (72.2)	132 (60.6)	53 (75.7)	7.0, 2	0.029
Missing	20 (27.8)	86 (39.5)	17 (24.3)		
Contact with family					
Present	54 (75.0)	132 (60.6)	55 (78.6)	10.4, 2	0.005
Missing	18 (25.0)	86 (39.5)	15 (21.4)		
Close confidants					
Present	59 (81.9)	159 (72.9)	59 (84.3)	5.1, 2	0.077
Missing	13 (18.1)	59 (27.1)	11 (17.7)		
Life events					
Present	21 (29.2)	34 (15.6)	15 (21.4)	6.6, 2	0.037
Missing	51 (70.8)	184 (84.4)	55 (78.6)		
Childhood adversity					
Present	33 (45.8)	70 (32.1)	34 (48.6)	8.4, 2	0.015
Missing	39 (54.2)	148 (67.9)	36 (41.4)		
Family history of any mental illness					
Present	58 (80.6)	143 (65.6)	58 (82.9)	11.13, 2	0.004
Missing	14 (19.4)	75 (34.4)	12 (17.1)		
Family history of psychosis					
Present	58 (80.6)	143 (65.6)	58 (82.9)	11.13, 2	0.004
Missing	14 (19.4)	75 (34.4)	12 (17.1)		
NSS					
Present	46 (63.9)	94 (43.1)	51 (72.9)	23.05, 2	<0.001
Missing	26 (36.1)	124 (56.9)	19 (27.1)		
MPAs					
Present	43 (59.7)	80 (36.7)	49 (70.0)	28.70, 2	<0.001
Missing	29 (40.3)	138 (63.3)	21 (30.0)		

*df* degrees of freedom, *MPA* minor physical abnormalities, *NSS* neurological soft signs, *PMD* psychotic major depression

### Findings

Table 4 shows the adjusted odds ratios for the association between each risk factor and each diagnostic group calculated with controls as the reference group, based on follow-up diagnosis. The table shows that living alone (aOR = 2.26, CI = 1.21–4.23), basic level qualification (aOR = 2.89, CI = 1.08–7.74), being unemployed (aOR = 2.12, CI = 1.13–3.96), having contact with friends less than monthly (aOR = 4.24, CI = 1.62–11.14), having no close confidants (aOR = 4.71, CI = 2.08–10.68), having experienced childhood adversity (aOR = 2.57, CI = 1.02–6.44), family history of mental illness (aOR = 10.68, CI = 5.06–22.52), family history of psychosis (aOR = 12.85, CI = 5.24–31.51), and having more neurological soft signs (aOR = 1.15, CI = 1.07–1.24) had large effect sizes and were statistically associated with a diagnosis of PMD. Having a severe life event in the year prior to illness onset had a substantial effect size (aOR = 3.32, CI = 0.96–11.45) but did not quite meet statistical significance (*p* = 0.06).

**Table 4:** Adjusted odds ratios adjusted for gender, age, centre and ethnicity and 95 % CIs for follow-up diagnosis of PMD, schizophrenia and bipolar compared with controls

	PMD vs. controls			Schizophrenia vs. controls			Bipolar vs. controls		
	aOR	95 % CI	*P*	aOR	95 % CI	*P*	aOR	95 % CI	*P*
Place of birth (*n* = 734)									
UK	1.00	–	–	1.00	–	–	1.00	–	–
Non-UK	1.00	0.40–2.51	0.995	0.96	0.56–1.65	0.886	0.55	0.22–1.36	0.197
Relationship status (n726)									
Stable relationship	1.00	–	–	1.00	–	–	–	–	–
Single	1.69	0.91–3.13	0.096	5.36	3.46–8.28	<0.001	2.03	1.16–2.57	0.014
Ever had a long-term relationship (n643)									
Yes	1.00	–	–	1.00	–	–	1.00	–	–
No	1.67	0.69–4.04	0.255	4.08	2.51–6.63	<0.001	2.08	1.04–4.16	0.038
Living with (n735)									
With people	1.00	–	–	1.00	–	–	1.00	–	–
Alone	2.26	1.21–4.23	0.011	2.93	1.97–4.37	<0.001	1.27	0.71–2.28	0.426
Level of Education (n729)									
Higher	1.00	–	–	1.00	–	–	1.00	–	–
Further	1.68	0.57–4.93	0.347	1.65	0.90–3.04	0.107	1.18	0.57–2.45	0.658
Basic	2.89	1.08–7.74	0.035	3.34	1.91–5.84	<0.001	0.98	0.48–1.98	0.946
Employment Status (n729)									
Employed and other	1.00	–	–	1.00	–	–	1.00	–	–
Unemployed	2.12	1.13–3.96	0.019	4.33	2.87–6.53	<0.001	1.58	0.94–2.64	0.082
Contact with friends (n608)									
Daily–monthly	1.00	–	–	1.00	–	–	1.00	–	–
Never/less than monthly	4.24	1.62–11.14	0.003	12.59	6.66–23.78	<0.001	1.77	0.57–5.44	0.321
Contact with family (n604)									
Daily–monthly	1.00	–	–	1.00	–	–	1.00	–	–
Never/less than monthly	1.83	0.34–9.94	0.482	5.58	2.13–14.60	<0.001	1.05	0.13–8.50	0.965
Close confidants (n655)									
Yes	1.00	–	–	1.00	–	–	1.00	–	–
No	4.71	2.08–10.68	<0.001	11.32	6.37–20.10	<0.001	2.72	1.14–6.48	0.024
Severe Life Events (n215)									
No	1.00	–	–	1.00	–	–	1.00	–	–
Yes	3.32	0.96–11.45	0.058	2.86	0.97–8.44	0.056	5.56	1.97–15.71	0.001
Childhood Adversity (n378)									
No	1.00	–	–	1.00	–	–	1.00	–	–
Yes	2.57	1.02–6.44	0.045	2.97	1.41–6.25	0.004	1.55	0.65–3.71	0.323
Family history of any mental illness (n641)									
No	1.00	–	–	1.00	–	–	1.00	–	–
Yes	10.68	5.06–22.52	<0.001	6.96	4.10–11.84	<0.001	13.19	6.64–26.20	<0.001
Family history of psychosis (n641)									
No	1.00	–	–	1.00	–	–	1.00	–	–
Yes	12.85	5.24–31.51	<0.001	10.16	5.37–19.25	<0.001	8.67	3.87–19.44	<0.001
NSS (n306)	1.15	1.07–1.24	<0.001	1.19	1.11–1.28	<0.001	1.15	1.07–1.23	<0.001
MPAs (n288)	1.10	0.97–1.24	0.123	1.27	1.16–1.41	<0.001	1.24	1.11–1.38	<0.001

*CI* confidence interval, *df* degrees of freedom, *MPAs* minor physical abnormalities, *NSS* neurological soft signs, *PMD* psychotic major depression

Table 4 also shows that all of these risk factors were associated with a diagnosis of schizophrenia. Additionally, being single (aOR = 5.36, CI = 3.46–8.28) and never having had a long-term relationship (aOR = 4.08, CI = 2.51–6.63) were associated with schizophrenia (but not PMD). Only some of the variables associated with PMD were also associated with a diagnosis of bipolar disorder, with having no close confidants (aOR = 2.72, CI = 1.14–6.48), having a family history of mental illness (aOR = 13.19, CI = 6.64–26.20), having a family history of psychosis (aOR = 8.67, CI = 3.87–19.44), and having more neurological soft signs (aOR = 1.15, CI = 1.07–1.23) associated with both PMD and bipolar disorder. Interestingly, minor physical anomalies were associated with a follow-up diagnosis of schizophrenia (aOR = 1.27, CI = 1.16–1.41) and bipolar disorder (aOR = 1.24, CI = 1.11–1.38), but not with PMD (aOR = 1.10, CI = 0.97–1.24). Being single and never having had a long-term relationship were associated with follow-up diagnosis of schizophrenia (aOR = 5.36, CI = 3.46–8.28 and aOR = 4.08, CI = 2.51–6.63, respectively) and psychotic bipolar disorder (aOR = 2.03, CI = 1.16–2.57 and aOR = 2.08, CI = 1.04–4.16, respectively). Variables associated with all three diagnoses were: no close confidants; family history of any mental illness; family history of psychosis; and NSS.

These analyses were repeated based on baseline diagnoses to examine differences in findings related to diagnostic change (see online appendix). For PMD cases, basic level education, contact with friends less than monthly, having no close confidants, having a family history of mental illness, a family history of psychosis, and more neurological soft signs remained significant with large effect sizes.

Being single, having experienced a severe life event in the year prior to onset and having minor physical anomalies became statistically significant and had a larger effect size but the direction of the effect stayed the same, while the effect size for having contact with family less than monthly increased but did not quite meet statistical significance. Living alone, being unemployed and having experienced childhood adversity had reduced effect sizes and became non-statistically significant but the direction of the effects stayed the same.

For schizophrenia, there were very few differences between risk factors associated with schizophrenia in the baseline and follow-up diagnosis analyses. The only substantial difference was that having contact with family less than monthly was not statistically significant in the baseline analyses.

For bipolar disorder, the only differences were living alone, contact with friends less than monthly, and being unemployed were associated with a baseline diagnosis of bipolar disorder.

## Discussion

### Main finding

As follow-up diagnoses in this study are based on far more information, and previous research has indicated that initial diagnoses are likely to change over time [25], we assumed that follow-up diagnoses were more reliable compared with baseline diagnosis and thus focused on the former in the analyses. First, in terms of psychosocial risk factors, there was more overlap between schizophrenia and PMD than between PMD and bipolar disorder. This is particularly noteworthy because some cases of PMD are often assumed to be bipolar disorder which has not yet manifested in a manic episode and some studies report PMD being most likely to change to a diagnosis of bipolar disorder over time [19]. Findings from this study indicate that PMD could be more like schizophrenia than previously thought, and that psychosocial factors are less relevant in bipolar disorder. There are a number of possible explanations of the similarities in risk factors between PMD and schizophrenia. First, this could be due to misdiagnosis spawning from difficulties in recognising depressive symptoms in patients with florid psychotic symptoms or misinterpretation of negative symptoms as depressive symptoms. Alternatively, if the similarities in risk factors between the two diagnostic groups are correct, this could indicate that PMD and schizophrenia are more closely related than previously recognised. Further work on shared and distinct risk factors for both disorders—including genetic and neuroimaging data—will help to further clarify this.

In terms of biological indicators, family history of mental illness and psychosis, and neurological soft signs were associated with PMD, schizophrenia and bipolar disorder, but minor physical anomalies were only associated with schizophrenia and bipolar disorder. This is consistent with previous literature which has found an association between minor physical anomalies and schizophrenia [22, 23, 35]. This could possibly indicate that there is a biological commonality between schizophrenia and bipolar which is not present in PMD.

The same set of risk factors is associated with PMD when using the baseline versus follow-up diagnoses in terms of direction and approximate size of effect, but the difference is in the statistical significance of each risk factor. This difference could be to do with diagnostic stability and the baseline diagnosis being unreliable. However, as most variables have the same direction of effect but the statistical significance has changed, this is likely to be a result of reduced power.

A recent study has reported that associations between childhood trauma and depression, mania, anxiety and psychosis were comparable, but that there was a much stronger association between childhood trauma and patients experiencing symptoms in multiple domains [44]. Findings from the current study do not support this increased association in PMD and bipolar cases who are essentially experiencing a combination of mood and psychotic symptoms. However, this study did not account for symptoms domains, only main clinical diagnosis, alongside which, other symptoms are commonly experienced (e.g. depressive symptoms are common in patients with a diagnosis of schizophrenia). Examining specific symptoms may have revealed a similar association to that reported by van Nierop et al. [44].

### Strengths and limitations

The most notable limitation was the potential for selection bias. There were significant differences in missing data between cases and controls. This difference in missing data is common in studies of this type, where controls volunteered to participate and a replacement control was found when a control refused participation. Cases, on the other hand, were selected due to their presentation to mental health services within defined geographical locations with a first episode of psychosis and an alternative could not be obtained. Further, it is difficult to collect data from patients experiencing their first psychotic episode as it is often a very distressing time and patients may be reluctant to talk to researchers who they have just met. However, there were also significant differences between diagnostic groups in a number of variables. This could have introduced bias into the study. For example, with variables such as life events and childhood adversity, cases with these experiences may refuse to answer questions on these topics as it is too distressing. Of note, less data were available for schizophrenia which may mean that the occurrence of life events and childhood adversity might have been underestimated in this group. Therefore, caution is needed in the interpretation of any results given the patterns of missing data.

Self-report data are liable to recall and interviewer bias. However, interviews were based on standardised, established questionnaires, were conducted by trained interviewers, and were administered to cases and controls in the same way to reduce interviewer bias. Data from clinical records are liable to recording biases and are not available for controls. Furthermore, recall bias is likely to be the same across all diagnoses so should not have led to biased differences between the diagnostic groups.

Within this study, adjustment for key demographic variables was conducted. It is possible that associations exist among some of the risk factors which would require adjustment for each other, e.g. childhood adversity has been found to be associated with life events [4, 5, 38]. Further, there is possibly cumulative effect of having multiple risk factors. However, due to low numbers, it was not sensible to control for associations between the other risk factors, or to examine cumulative effect as this would seriously reduce the power of the analyses. This is something that would best be examined in another study with a larger sample size. However, this was the first step in identifying risk factors for PMD and future causal research could be used to examine the impact of certain risk factors while controlling for others. The high number of statistical comparisons means that multiple testing is an issue in this study and that a number of associations are likely due to chance. Finally, imprecision is an important limitation as the confidence intervals of some of the estimates are very wide and therefore, some of the ‘medium’ magnitude effects could be quite close to the null even though they are statistically significant.

The confidence intervals are very wide; theoretically, some of the ‘medium’ magnitude effects could be quite close to the null even though they are statistically significant by conventional cut-offs. So, imprecision is definitely important to mention.

Despite these limitations, this study contributes evidence beyond previous research through its use of a minimally biased sample than previously used, and through rigorous methodology around diagnosis (accounting for diagnostic change; consensus diagnoses made blind to ethnicity and baseline diagnoses). Furthermore, although the variables used to investigate risk factors were fairly crude (family history could indicate genetic or environmental factors) and biological indicators (neurological soft signs and minor physical anomalies) were less advanced than more innovative techniques such as neuroimaging, this is the first study to examine risk factors for PMD across a wide range of domains and to try to examine specificity of diagnosis.

### Findings and implications

The finding that the psychosocial risk factors investigated were not unique to PMD has several plausible interpretations. These are: (1) these psychosocial factors have been measured with insufficient precision to find the specifying factors (e.g. variables are oversimplified, such as in a relationship versus not); (2) the psychosocial factors investigated pose a risk generic for all psychoses, and it is other psychosocial factors that are the specifying factors (e.g. higher level psychosocial factors such as urban density); (3) the psychosocial factors investigated are generic in the risk for psychosis and it is some other factor, e.g. genetic factors, that are the specifying factors; (4) the diagnostic classification system used does not sufficiently distinguish between different disorders which confuse the aetiological picture. Based on data presented here, it is not possible to determine which interpretation is correct.

## Conclusion

Further work on the differential aetiology of PMD, from other psychoses, is needed to find the ‘specifier’ between PMD and other psychoses. Future research on aetiology in PMD, and perhaps other psychoses, should account for diagnostic stability.

## Electronic supplementary material

Supplementary material 1 (DOCX 27 kb)
