# Supplementary material for: Biological and psychosocial risk factors for psychotic major depression
Source: Soc Psychiatry Psychiatr Epidemiol. 2015 Oct 31;51:233–45. doi: 10.1007/s00127-015-1131-1 (PMC4748002; doi:10.1007/s00127-015-1131-1)
Supplement: Supplementary file 1 — Supplementary material 1 (DOCX 27 kb) [file 127_2015_1131_MOESM1_ESM.docx]

**Supplementary tables – online appendix**

**Adjusted odds ratios adjusted for gender, age, centre and ethnicity and 95% CIs for baseline diagnosis of PMD, schizophrenia and bipolar compared with controls**

|  | **PMD vs. controls** | | | **Schizophrenia vs. controls** | | | **Bipolar vs. controls** | | |
| --- | --- | --- | --- | --- | --- | --- | --- | --- | --- |
|  | **Adjusted OR** | **95% CI** | **P** | **Adjusted OR** | **95% CI** | **P** | **Adjusted OR** | **95% CI** | **P** |
| Place of birth (n744):  UK  Non-UK | 1.0  1.20 | -  0.53-2.72 | -  0.667 | 1.0  0.93 | -  0.55-1.57 | -  0.787 | 1.0  0.55 | -  0.23-1.31 | -  0.176 |
| Relationship Status (n736):  Stable relationship  Single | 1.0  2.05 | -  1.19-3.53 | -  0.009 | 1.0  5.05 | -  3.29-7.75 | -  <0.001 | 1.0  2.07 | -  1.16-3.69 | -  0.014 |
| Ever had a long term relationship (n653):  Yes  No | 1.0  1.67 | -  0.79-3.50 | -  0.178 | 1.0  3.37 | -  2.10-5.41 | -  <0.001 | 1.0  2.54 | -  1.27-5.08 | -  0.008 |
| Living with (n747):  With people  Alone | 1.0  1.41 | -  0.81-2.47 | -  0.222 | 1.0  2.44 | -  1.64-3.63 | -  <0.001 | 1.0  2.34 | -  1.35-4.06 | -  0.003 |
| Level of Education (n738):  Higher  Further  Basic | 1.0  0.98  2.42 | -  0.39-2.48  1.07-5.43 | -  0.967  0.033 | 1.0  1.66  3.01 | -  0.93-2.99  1.44-5.12 | -  0.088  <0.001 | 1.0  1.16  0.95 | -  0.55-2.43  0.46-1.99 | -  0.692  0.894 |
| Employment Status (n740):  Employed and other  Unemployed | 1.0  1.68 | -  0.98-2.86 | -  0.057 | 1.0  3.44 | -  2.30-5.15 | -  <0.001 | 1.0  2.24 | -  1.31-3.85 | -  0.003 |
| Contact with friends (n615):  Daily – monthly  Never / less than monthly | 1.0  4.17 | -  1.71-10.17 | -  0.002 | 1.0  10.89 | -  5.88-20.18 | -  <0.001 | 1.0  2.99 | -  1.18-7.59 | -  0.021 |
| Contact with family (n609):  Daily – monthly  Never / less than monthly | 1.0  3.15 | -  0.88-11.25 | -  0.077 | 1.0  2.77 | -  0.97-7.94 | -  0.058 | 1.0  1.70 | -  0.33-8.84 | -  0.526 |
| Close confidants (n665):  Yes  No | 1.0  4.45 | -  2.11-9.41 | -  <0.001 | 1.0  10.93 | -  6.24-19.16 | -  <0.001 | 1.0  4.85 | -  2.30-10.20 | -  <0.001 |
| Life Events (n217):  No  Yes | 1.00  5.25 | -  1.64-16.82 | -  0.005 | 1.0  2.17 | -  0.77-6.14 | -  0.144 | 1.0  3.94 | -  1.19-13.03 | -  0.025 |
| Childhood Adversity (n379):  No  Yes | 1.0  1.30 | -  0.58-2.94 | -  0.522 | 1.0  6.42 | -  2.71-15.23 | -  <0.001 | 1.0  1.36 | -  0.60-3.11 | -  0.459 |
| Family history of any mental illness (n650):  No  Yes | 1.0  5.49 | -  2.70-11.16 | -  <0.001 | 1.0  7.77 | -  4.53-13.32 | -  <0.001 | 1.0  10.90 | -  5.44-21.85 | -  <0.001 |
| Family history of psychosis (n650):  No  Yes | 1.0  5.83 | -  2.36-14.38 | -  <0.001 | 1.0  11.00 | -  5.76-21.00 | -  <0.001 | 1.0  7.87 | -  3.41-18.13 | -  <0.001 |
| NSS (n317) | 1.18 | 1.10-1.22 | <0.001 | 1.18 | 1.10-1.27 | <0.001 | 1.13 | 1.04-1.22 | 0.002 |
| MPAs (n296) | 1.17 | 1.05-1.30 | 0.005 | 1.24 | 1.13-1.37 | <0.001 | 1.19 | 1.07-1.31 | 0.001 |

CI, confidence interval. Df, degrees of freedom. MPAs, minor physical abnormalities. NSS, neurological soft signs. PMD, psychotic major depression
